# Supplementary material for: Centering healthcare workers in developing digital health interventions: usability and acceptability of a two-way texting retention intervention in a public HIV clinic in Lilongwe, Malawi
Source: medRxiv. 2023 Jan 11:2023.01.09.23284326. Preprint. [Version 1] doi: 10.1101/2023.01.09.23284326 (PMC9882492; doi:10.1101/2023.01.09.23284326)
Supplement: Supplement 1 [file NIHPP2023.01.09.23284326v1-supplement-1.pdf]

medRxiv preprint doi: https://doi.org/10.1101/2023.01.09.23284326; this version posted January 11, 2023. The copyright holder for this preprint (which was not certified by peer review) is the author/funder, who has granted medRxiv a license to display the preprint in perpetuity.
It is made available under a CC-BY 4.0 International license .

Supporting information

S1 Figure 1. Developing and optimizing 2wT for ART retention: Medic's human-centered design process [16] [REDACTED]

medRxiv preprint doi: https://doi.org/10.1101/2023.01.09.23284326; this version posted January 11, 2023. The copyright holder for this preprint (which was not certified by peer review) is the author/funder, who has granted medRxiv a license to display the preprint in perpetuity.
It is made available under a CC-BY 4.0 International license .

S2 Figure 2. 2wT flow diagram for ART retention

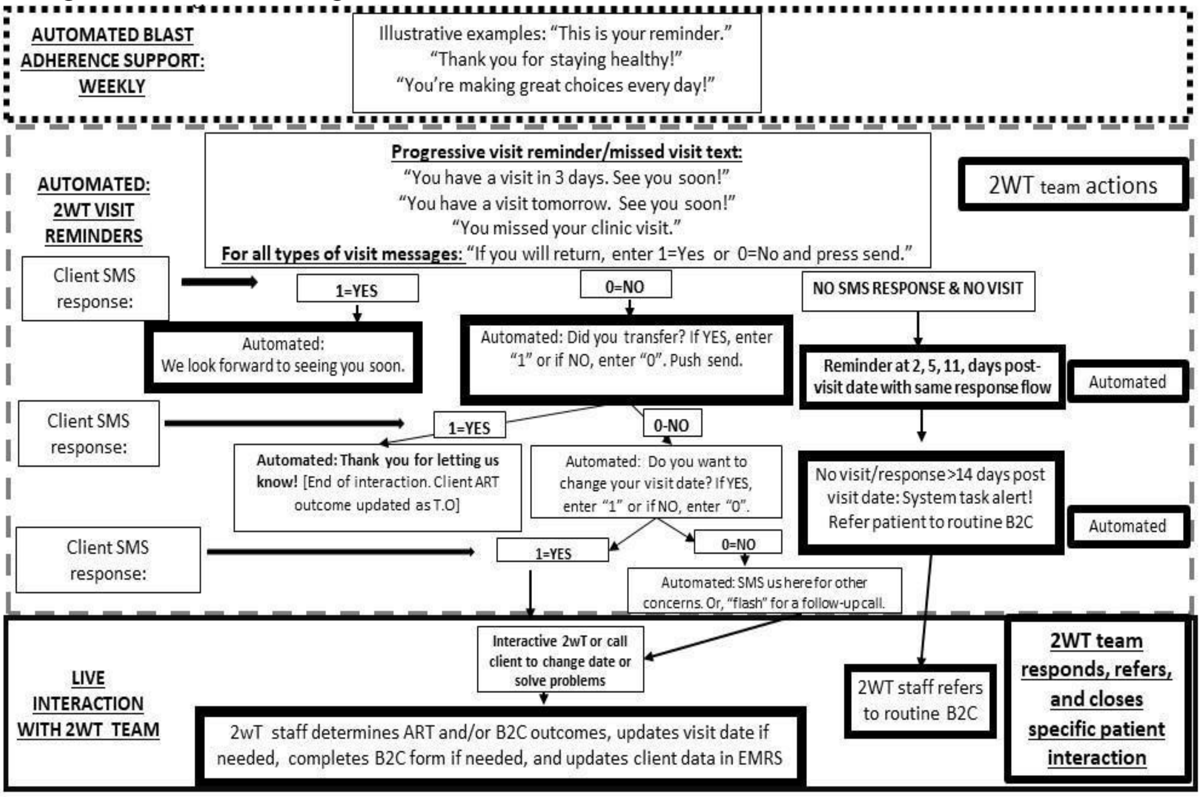

medRxiv preprint doi: https://doi.org/10.1101/2023.01.09.23284326; this version posted January 11, 2023. The copyright holder for this preprint (which was not certified by peer review) is the author/funder, who has granted medRxiv a license to display the preprint in perpetuity.
It is made available under a CC-BY 4.0 International license .

S3 Figure 3. 2wT actions completed by HCWs

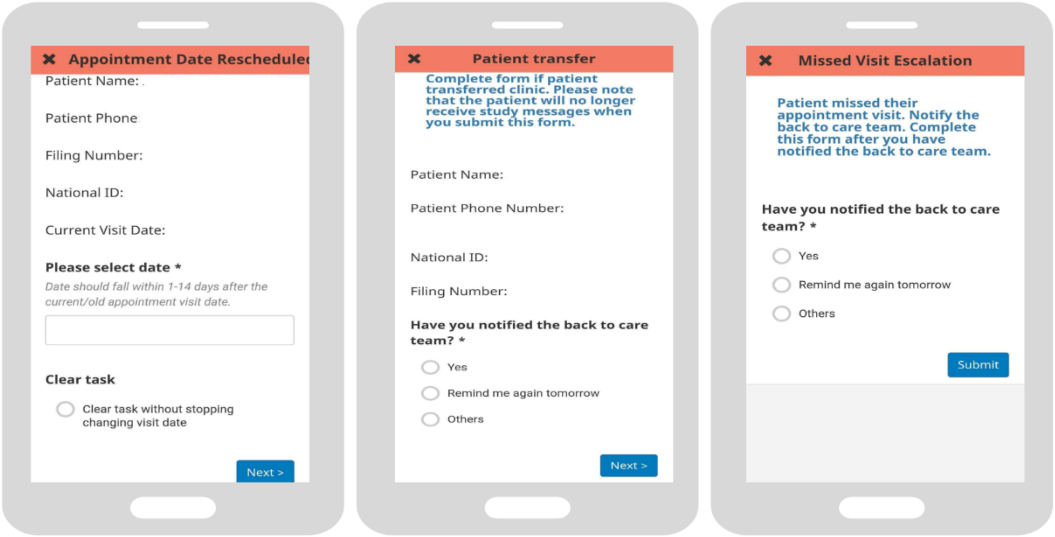

medRxiv preprint doi: https://doi.org/10.1101/2023.01.09.23284326; this version posted January 11, 2023. The copyright holder for this preprint (which was not certified by peer review) is the author/funder, who has granted medRxiv a license to display the preprint in perpetuity.
It is made available under a CC-BY 4.0 International license .

S1 Table. Emergent themes

THEME	DEFINITION
**MAJOR**	
Potential reduced workload	Reduced workload for the B2C team
Adherence benefits for clients	Support for clients to maintain adherence
Privacy issues for clients	Clients feel someone can see the messages and figure it it's regarding ART
2wT excludes some clients	Some clients who would benefit from 2wt are ineligible to enrol
Technology issues	System or network connectivity issues affecting the 2wt platform or electronic medical records system (EMRS)
Strengthen 2wT education for clients	Strengthen client understanding of the 2wT messages and usage
**MINOR**	
Address 2wT interoperability/tech concerns	Address 2wT technical concerns regarding 2wT interoperability with the EMRs
Review cost considerations	Review cost considerations of the SMS platform
Supportive motivational messaging for clients	Motivational messaging strengthened client- health provider relationship
Diversify messaging to include healthy behaviors	Include other healthy living behaviors that support adherence
